# Quantitative Assessment of GFAP-Based Astrocyte Morphology in the Cuprizone Model: A Comparative Evaluation of Neurolucida^®^ 360 and SNT

**DOI:** 10.3390/cells15110964

**Published:** 2026-05-22

**Authors:** Lukas Wenzel, Leo Heinig, Dongshi Wang, Elise Vankriekelsvenne, Nicole Wigger, Annelie Zimmermann, Johann Rößler, Tim Clarner, Markus Kipp

**Affiliations:** 1 Institute of Anatomy, Rostock University Medical Center, Gertrudenstr. 9, 18057 Rostock, Germany; 2 Institute of Pathology, University Medical Center of the Johannes Gutenberg University Mainz, Langenbeckstraße 1, 55131 Mainz, Germany; 3 Anatomy and Cell Biology, University Hospital Bonn, University of Bonn, Nussallee 10, 53115 Bonn, Germany

**Keywords:** astrocytes, reactive gliosis, GFAP, cuprizone model, Neurolucida^®^ 360, SNT, morphology, digital reconstruction, hippocampus, multiple sclerosis

## Abstract

Reactive astrocytes are a hallmark of several neurological diseases in multiple sclerosis and experimental demyelination models. Their morphological alterations are commonly assessed by qualitative histopathology, yet quantitative tools are required to better capture astrocytic heterogeneity and to allow correlations with imaging-derived biomarkers. Here, we present a workflow for the quantitative analysis of Glial Fibrillary Acidic Protein (GFAP) network remodeling in astrocytes in the cuprizone model of demyelination. C57BL/6 mice were intoxicated with cuprizone for 3 or 5 weeks to induce progressive demyelination, microglial activation, and reactive astrogliosis. Brain sections were processed for anti-GFAP immunohistochemistry, and individual astrocytes from the stratum oriens of the hippocampus were digitally reconstructed. Diverse parameters of GFAP topology, including soma size, process length, branching order, convex hull area, and ramification index, were extracted using either the commercial Neurolucida^®^ 360 software or the open-source Simple Neurite Tracer (SNT) plugin in ImageJ. Principal component analysis revealed clear differences between control astrocytes and astrocytes in cuprizone-intoxicated animals, with reactive astrocytes displaying increased numbers of primary processes, enhanced bifurcation, and process complexity. Comparative evaluation of Neurolucida^®^ 360 and SNT demonstrated that both tools are suitable for astrocyte reconstruction, although Neurolucida^®^ 360 enabled faster and more detailed tracing. This protocol provides a reproducible pipeline for the quantitative assessment of astrocyte morphology under control and pathological conditions, thereby supporting future efforts to link cellular remodeling to functional outcomes in neuroinflammatory disease models.

## 1. Introduction

Multiple sclerosis (MS) is a chronic inflammatory disease of the central nervous system (CNS) and one of the most prevalent neurological disorders worldwide, with an increasing incidence [1,2]. This disease manifests with a broad spectrum of clinical symptoms, including sensory, visual, cognitive, and motor deficits. On the histological level, inflammatory demyelinating lesions can be found throughout the white and gray matter, including the hippocampus formation [3,4,5,6,7,8]. Besides demyelination and axonal damage [9,10,11], microglial activation, oligodendrocyte degeneration, and lymphocyte invasion are histopathological hallmarks [12,13]. As the disease progresses, histopathological lesions evolve from an active inflammatory state toward an inactive, chronic stage [14]. This lesion progression is accompanied by the formation of glial scars—structures composed predominantly of reactive astrocytes—which serve to isolate damaged tissue but may concurrently impede remyelination and axonal regeneration within MS lesions [15,16].

Astrocytes are a major subtype of CNS glial cells, encompassing specialized forms such as Bergmann glia in the cerebellum or Müller glia in the retina [17,18]. Far from being merely supportive, astrocytes perform a wide array of essential tasks that are critical for CNS homeostasis and neural function. For example, astrocytes are key structural and functional components of the blood–brain barrier (BBB), where their perivascular endfeet ensheath blood vessels and capillaries, thus contributing to the regulation of cerebral blood flow and barrier integrity [19]. Astrocytes also provide vital metabolic support to neurons by shuttling nutrients and maintaining ionic balance, especially through the uptake and recycling of neurotransmitters like glutamate [20]. In addition, astrocytes maintain intimate contact with synapses, forming so-called “tripartite synapses,” where they not only monitor but actively modulate synaptic transmission. This is achieved via intracellular Ca^2+^ (calcium ion) signaling and the release of gliotransmitters such as ATP (adenosine triphosphate) and D-serine, which influence synaptic strength and plasticity [21,22]. Through these and other mechanisms, astrocytes play a pivotal role in shaping neural networks and information processing.

Under pathological conditions—such as CNS trauma, stroke, infection, or chronic neuroinflammation—astrocytes undergo profound phenotypic and functional alterations collectively termed reactive astrogliosis [23]. Reactive astrogliosis is accompanied by extensive transcriptional reprogramming, as well as changes in calcium signaling, glutamate uptake, and cytokine secretion [24]. From a functional perspective, this process is characterized by a context-dependent shift from homeostatic support toward phenotypes that can exert either detrimental or beneficial effects on neural tissues [25]. For example, it has been shown that reactive astrocytes might promote the survival of regenerating oligodendrocytes through downregulation of the transcription factor Nrf2 pathway [26], can protect hippocampal neurons against ischemia by suppressing the intracellular Ca^2+^ overload [27], and are capable of protecting dopaminergic neurons from α-synuclein accumulation and propagation [28]. On the other hand, it has been demonstrated that astrocytes can mediate synapse elimination through MEGF10 (Multiple Epidermal Growth Factor-Like Domains Protein 10) and MERTK (MER Proto-Oncogene, Tyrosine Kinase) pathways [29] or can provoke synaptic loss through the augmented release of glutamate [30].

On the histological level, reactive astrocytes also undergo clear morphological alterations. From a historical perspective, “resting” astrocytes were categorized into protoplasmic and fibrous types. Fibrous astrocytes are typically found in the white matter and possess long, thin processes, whereas protoplasmic astrocytes are in the gray matter and exhibit thicker, shorter, and highly bifurcated processes [31,32,33]. More sophisticated methods, such as the guided expression of fluorescence proteins or the injection of fluorescence dyes into individual astrocytes, displayed a characteristic “bushy” morphology of astrocytes, which occupy defined territories in the nervous tissue [34,35].

In neuroinflammatory diseases such as MS, astrocytes undergo reactive astrogliosis, involving pronounced morphological changes. Astrocytes in active MS lesions become markedly hypertrophic, with enlarged cell bodies and thickened processes, often accompanied by fewer fine processes indicative of severe tissue injury [36]. Beyond, reactive astrocytes retract their perivascular endfeet, compromising BBB integrity and, therefore, eventually facilitating immune cell infiltration [36,37]. These and other structural transformations presumably have important implications for disease progression in MS patients. Crucial for the morphological adaptations of astrocytes are dynamic changes in the cytoskeleton.

The cytoskeleton is a dynamic network of protein filaments that provides structural support, maintains cell shape, and facilitates cellular movement and intracellular transport. It consists of three main components: microfilaments (actin filaments), which are thin (~7 nm in diameter) and contribute to cell shape, motility, and muscle contraction; microtubules, which are hollow tubes (~25 nm in diameter) serving as tracks for intracellular transport and playing key roles in cell division and organelle positioning; and intermediate filaments, rope-like fibers (~10 nm in diameter) that provide mechanical strength and maintain structural integrity, especially under mechanical stress. Intermediate filaments are more stable than microfilaments and microtubules and do not exhibit dynamic polymerization [38,39,40,41]. Instead, they form a dense network throughout the cytoplasm and are anchored to the nuclear envelope and cell–cell junctions (desmosomes). In astrocytes, Glial Fibrillary Acidic Protein (GFAP) is the major type of intermediate filament protein and is commonly used as a marker of astroglial identity and reactivity in neuropathological conditions. As stated above, one of the hallmark changes in astrocytes under neuroinflammatory conditions is hypertrophy [42]. This is accompanied by a reorganization of the cytoskeleton, particularly of intermediate filaments such as GFAP and vimentin, which accumulate in reactive astrocytes and contribute to their altered shape and rigidity [43]. The importance of the intermediate filament system in reactive astrocytes is further demonstrated by the finding that *Gfap*^−/−^*Vimentin*^−/−^ mice show reduced reactive gliosis and glial scarring, paralleled by slower healing with an increased loss of neuronal synapses following neurotrauma [44,45]. A deeper understanding of intermediate filament alterations during reactive gliosis is crucial for elucidating their functional significance.

Different animal models, such as the cuprizone model, exist to investigate cellular alterations during neuroinflammation. The cuprizone model, a toxin-induced demyelination paradigm, involves administration of the copper chelator cuprizone (bis(cyclohexanone)oxaldihydrazone), which triggers oligodendrocyte apoptosis and subsequent demyelination [46]. This is accompanied by microglial activation and reactive astrogliosis, thus providing a robust system to study astrocyte morphology and function in vivo [47,48,49].

In this study, we aimed to develop a pipeline to quantify changes in GFAP expression topology in resting and activated astrocytes. To this end, paraffin sections from control and cuprizone-intoxicated mice were processed for anti-GFAP immunohistochemistry (IHC), and cellular models were generated using either the commercially available software tool Neurolucida^®^ 360 [50] or the open source software ImageJ package SNT (Simple Neurite Tracer) [51,52].

## 2. Materials and Methods

### 2.1. Experimental Animals and Wet-Lab Procedures

For this project, fifteen 9-week-old female C57BL/6 mice were purchased from Janvier Labs (Le Genest-Saint-Isle, France). The animals were randomly assigned to three cages (5 mice per cage). This assignment was carried out by an animal caretaker who was not further involved in the study. The three cages represent the experimental groups: the control group (Cntrl), 3 weeks cuprizone (3Wks.Cup) and 5 weeks cuprizone (5Wks.Cup). The animals were housed under standard laboratory conditions (23 ± 2 °C with a 12 h light/dark cycle) with ad libitum access to food and water. Daily health checks were performed, and cages were changed three times per week. Microbiological monitoring was performed according to the recommendations of the Federation of European Laboratory Animal Science Associations (FELASA). The experiment was approved by the Animal Care and Use Committee of the Mecklenburg-Western Pomerania district government (reference number 7221.3-1-062/21).

**Figure 1:**
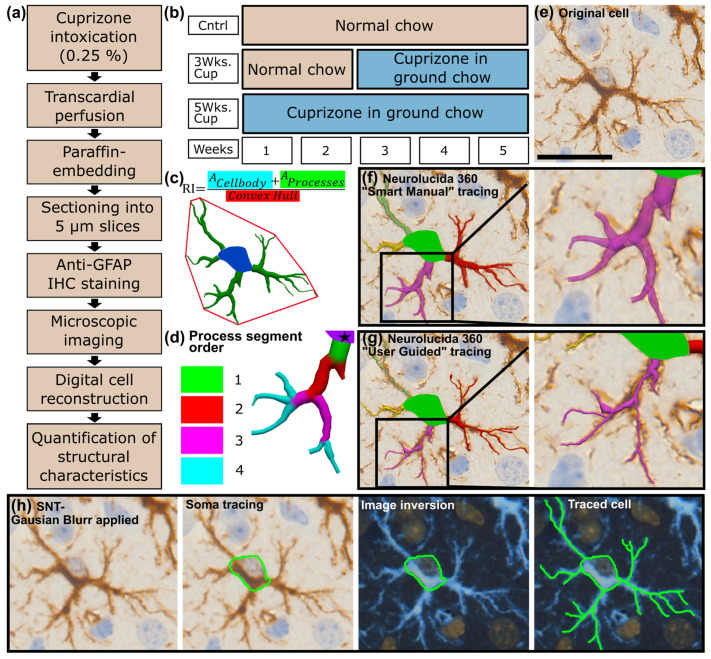
(**a**) Schematic illustration of the 3 experimental groups. (**b**) Schematic illustration of the experimental setup and workflow. (**c**) Principal of ramification index (RI) computation is shown with the total cell area (process area (green) + cell body area (blue)) and the convex hull area (CH, red). Note that highly ramified cells exhibit a ramification index far below 1, while non-ramified cells show values close to 1. (**d**) Partial reconstruction of an astrocyte using Neurolucida^®^ 360, visualized in the Neurolucida^®^ Explorer environment. By definition, first-order segments (highlighted in green) originate directly from the soma (highlighted by *) and terminate at the starting point of a second-order segment (highlighted in red). (**e**) High-power image of a single anti-GFAP-labeled astrocyte with nuclear counterstaining before reconstruction. (**f**) Digital cell models, which also take process width into account, were created with the “Smart Manual” tracing method in Neurolucida^®^ 360. (**g**) Cell model generated using the “user-guided” method in Neurolucida^®^ 360, accounting only for process length but not cell area. (**h**) A Gaussian blur was applied to images before cell reconstructions with the SNT, followed by soma tracing. Finally, the image was inverted and the processes traced. Scale bars = 25 µm.

To induce graded astrocyte activation, mice were intoxicated either for 3 or 5 weeks with cuprizone (0.25%), as published previously [53] (Figure 1b). To this end, 0.25 g of cuprizone was weighed using precision scales and mechanically mixed with 100 g of ground standard rodent chow using a commercially available kitchen machine (Kult X, WMF Group, Geislingen an der Steige, Germany). The chow was mixed at low speed and manual agitation for 1 min and was provided within the cage in 2 separate plastic petri dishes (100 g per cage). Following the respective intoxication periods, animals were euthanized, and brains were fixed by transcardial perfusion followed by immersion fixation. Tissues were embedded in paraffin, sectioned at 5 µm thickness, and processed for immunohistochemistry using antibodies against GFAP to visualize astrocytes. To assess demyelination and microglial activation, IHC stainings against PLP (Proteolipid Protein 1) and IBA1 (Ionized Calcium-Binding Adaptor Molecule 1) were carried out.

### 2.2. Quantification of Spatial Anti-GFAP Immunoreactivity

To evaluate spatial anti-GFAP immunoreactivity in a quantifiable manner at different stages of reactive astrogliosis, digital cell models were generated of individual astrocytes (Figure 1a). To ensure unbiased selection of the cells, a total of 150 anti-GFAP-labelled astrocytes (10 per animal, with 5 animals per experimental group) were randomly selected via computational methods for reconstruction (see protocol section).

The region of interest, where the evaluated cells were selected, was the stratum oriens of the rostral hippocampus corresponding to the Bregma coordinates −1.06 [54]. The hippocampus, as a prototypical archicortical structure, is composed of the dentate gyrus and the cornu ammonis (CA1–CA3). The dentate gyrus consists of 3 layers: the outer molecular layer containing dendrites of granule cells and interneurons, the compact granule cell layer with densely packed somata, and the polymorphic layer (hi-lus). The cornu ammonis exhibits a comparable laminar organization, with the pyramidal cell layer flanked towards the alveus by the stratum oriens and on the other side by the stratum radiatum and stratum lacuno-sum-moleculare. We selected this region of interest because (i) cuprizone-induced demyelination involves the hippocampus formation [49]; (ii) in contrast to the neocortex, astrocytes exhibit robust GFAP expression under both control and demyelinating conditions; and (iii) unlike in the white matter tract of the corpus callosum, astrocytic process orientation in this region is not strongly influenced by the highly aligned axonal fibers that run between the two hemispheres within the corpus callosum.

### 2.3. Determining Overall GFAP Expression

To determine overall GFAP expression, the fraction of GFAP^+^ area within the region of interest (ROI; i.e., the stratum oriens located above the pyramidal layer of the cornu ammonis) was quantified. For this purpose, brain sections were digitized using a slide scanner (Ocus^®^ 20, Grundium, Tampere, Finland). The ROIs were manually delineated, after which the blue (hematoxylin) channel was subtracted by color deconvolution [55]. The resulting images were converted into binary formats using automated thresholding, and the fraction of GFAP^+^ pixels was subsequently calculated. Image processing and quantitative analyses were performed using QuPath [version 0.5.1] and ImageJ [56,57].

In addition, GFAP^+^ astrocytes were counted, and their number per mm^2^ within the ROIs was determined.

### 2.4. Generation of Digital Models of GFAP Distribution in Individual Astrocytes

Various software solutions exist for quantifying the morphological characteristics of single cells. Among these, Neurolucida^®^ 360 is a commercially available tool, whereas the SNT plugin offers an open-source alternative. For digital model construction, the software packages Neurolucida^®^ 360 [MBF Bioscience, Version 2022.1.1] + Neurolucida^®^ Explorer [MBF Bioscience, Willistion, USA; Version 2024.1.1] and the ImageJ-based application toolbox [52] were used. Different cellular parameters were assessed. The number, length, and area of cellular processes were quantified according to branch order. The segment extending from the soma to the first branching point was defined as the primary (first-order) segment; branches arising from primary segments were classified as secondary, with higher-order branches defined accordingly (Figure 1c). In addition, the soma area and the convex hull area were calculated. The convex hull area, as described by Heppner et al., represents the projected area encompassing the entire cell (Figure 1d) [58]. Furthermore, a ramification index was computed as the ratio of total cell area to convex hull area. Finally, the number of process segments, their length and area according to the process order were subjected to principal component analysis (PCA) to assess astrocyte heterogeneity.

### 2.5. Comparison Between Neurolucida^®^ 360 and the SNT

To evaluate both software environments, a comparative test was conducted in which 5 test persons reconstructed 6 individual astrocytes, as described below. In Neurolucida^®^ 360, the user-guided tracing algorithm—excluding process area analysis—was used. All participants had no prior experience with either software. Each participant received identical training before performing the tracing task. Half of the participants began with Neurolucida^®^ 360, the other half with the SNT. The time required to reconstruct individual astrocytes was recorded, and average processing times per astrocyte and software were calculated. To ensure comparability, reconstruction quality was assessed by quantifying the total number of process segments, number of primary process segments, mean segment length and the highest process order to reflect the level of detail in the traced cells.

### 2.6. Reagents

- Anti-GFAP antibodies (RRID: AB_1209224, Abcam, Cambridge, UK, cat. no. ab68428).
- Anti-IBA1 antibodies (RRID: AB_8395049, FUJIFILM Wako Pure Chemical Corporation, Osaka, Japan, cat. no. 019-19741).
- Anti-PLP antibodies (RRID: AB_2237198, Bio-Rad AbD Serotec GmbH, Neurid, Germany, cat. no. MCA839G).
- Cuprizone ((bis(cyclohexanone)oxaldihyrazone (Sigma-Aldrich Inc., St. Louis, MO, USA, cat. no. 370-81-0).
- DAB+ Chromogen Kit (OriGene Technologies Inc., Rockville, MD, USA cat. no. C09-100).
- DePeX mounting medium (SERVA Electrophoresis, Heidelberg, Germany cat. no. 18243.02).
- Di-sodium hydrogen phosphate dihydrate (Na_2_HPO_4_ 2H_2_O) (Merck, Darmstadt, Germany, cat. no. 1.06580.100).
- Embedding cassette (Roth, Karlsruhe, Germany, cat. no. EE16.1).
- EnVision with HPR labeled Polymer™ (DAKO/Agilent Technologies, Santa Clara, CA, USA, cat. no. K4001 and K4003).
- Ethanol denatured (Merck, Darmstadt, Germany, cat. no.N1006.9025).
- Formaldehyde solution 37% (Roth, Karlsruhe, Germany, cat. no. CP10.3).
- Ground chow (Sniff Spezialdiäten GmbH, Soest, Germany, cat. no. V1530-000).
- Hydrochloric acid (1 M) (HCl) (Roth, Karlsruhe, Germany, cat. no. K025.1).
- Hydrogen Peroxide 35% (H_2_O_2_) (Merck, Darmstadt, Germany, cat. no. 1.08600.1000).
- Ketamine (Ketabel^®^) (Bela-Pharm GmbH & Co. KG, Vechta, Germany, cat. no. E1670071).
- Mayer’s Hemalaum solution (Merck, Darmstadt, Germany, cat. no. 1.09249.0500).
- Microscope Cover Glasses (Paul Marienfeld GmbH & Co. KG, Lauda-Königshofen, Germany, cat. no. 0101222).
- Normal Goat Serum (Merck, Darmstadt, Germany, cat. no. 3947392).
- Paraffin (Histosec™ pastilles) (Merck, Darmstadt, Germany, cat. no. K958345 09).
- Paraffin embedding mold (Peel-A-Way^®^) (Merck, Darmstadt, Germany, cat. no. 18646A-1).
- Potassium chloride (KCl) (Roth, Karlsruhe, Germany, cat. no. 6781.1).
- Potassium dihydrogen phosphate (KH_2_PO_4_) (Merck, Darmstadt, Germany, cat. no. 1.04873.1000).
- Rodent chow (Sniff Spezialdiäten GmbH, Soest, Germany, cat. no. V1534-000).
- Sodium chloride (NaCl) (Roth, Karlsruhe, Germany, cat. no. 3957.2).
- Sodium dihydrogen phosphate (NaH_2_PO_4_) (Merck, Darmstadt, Germany, 1.06346.1000).
- Sodium hydroxide solution (NaOH) (Roth, Karlsruhe, Germany, cat. no. 6785.1).
- Superfrost™ Adhesion Microscope Slides (Epredia, Kalamazoo, MI, USA cat. no. J1800AMNZ).
- Xylazin (Rompun 2%) (Elanco GmbH, Monheim, Germany, cat. no. (01)04007221031017).
- Xylene (Merck, Darmstadt, Germany, cat. no. 28975.462).

### 2.7. Equipment

- Atraumatic tweezers, 105 mm (VWR by Avantor, Radnor, PA, USA, cat. no. 232–0086).
- Camera (Leica DMC 6200, Leica, Wetzlar, Germany).
- Ocus^®^ 20 slide scanner (Grundium, Tampere, Finland).
- Kitchen mixer (Groupe SEB WMF Retail GmbH, Geislingen/Stiege, Germany, cat. no. 4211129132435).
- Microscope (Leica DM6, Leica, Wetzlar, Germany).
- Microscopy scissors, 100 mm (VWR by Avantor, Radnor, PA, USA, cat. no. 233–1454).
- Microtome (Leica, RM225 Leica, Wetzlar, Germany).
- Microwave (Panasonic, Kadoma, Japan, NN-K129M).
- Perfusion System (Labortechnick Glattbrug, Glattbrugg-Zürich, Switzerland Typ: IPC-4).
- Precision scale (A + P Instruments GmbH, Meschede, Germany, cat. no. GR-202-EC).
- Scale (Kern & Sohn GmbH, Balingen, Germany, cat. no. 4045761475403).

### 2.8. Software

- Neurolucida^®^ 360 (MBF Bioscience, Version 2022.1.1).
- Neurolucida^®^ Explorer (MBF Bioscience, Willistion, ND, USA; Version 2024.1.1).
- ImageJ (version 1.54k).
- Leica Application Suite X software [version 3.7.0.20979, 2019, Germany].
- R-studio [version 4.3.1 (2023-06-16 ucrt)].
- QuPath [version 0.5.1].

### 2.9. Reagent Setup

#### 2.9.1. NGS 5% 

Take 2.5 mL of normal goat serum (NGS) and mix it with 47.5 mL 1x PBS (phosphate-buffered saline). Transfer the diluted serum into 2 mL reaction tubes and store at −20 °C.

#### 2.9.2. DAB Working Solution

Combine 95 mL of DAB (3,3′-diaminobenzidine) substrate with 5 mL of DAB chromogen. Store the solution at 4 °C in the dark; it remains usable for up to 5 days.

#### 2.9.3. Cuprizone-Enriched Ground Chow

To prepare the daily dose for ten mice, weigh 197.5 g of ground chow and 2.5 g of cuprizone. Use a precision scale for the cuprizone and a standard scale for the ground chow. Blend both components with a kitchen mixer (see equipment section) at the highest speed for 1 min, manually agitating the whole machine during mixing. Transfer the mixture into Petri dishes before placing it in the cages. Prepare the mixture freshly every day.

#### 2.9.4. PBS (10×) 

Dissolve 400 g of NaCl, 10 g of KCl, 84 g of Na_2_HPO_4_ 2H_2_O, and 13.5 g of KH_2_PO_4_ in 4000 mL of distilled water. Stir until completely dissolved, then adjust the pH to 6.8 using NaOH or HCl. If the pH remains stable after 20 min, add distilled water to a final volume of 5000 mL.

#### 2.9.5. PBS (1×)

Dilute 500 mL of 10× PBS (phosphate-buffered saline) stock solution with 4300 mL of distilled water in a beaker. Adjust the pH to 7.4 using NaOH or HCL. If the pH remains stable after 20 min, add distilled water to a final volume of 5000 mL.

#### 2.9.6. Perfusion Solution

Fill a beaker with 700 mL of distilled water and dissolve 4.6 g of NaH_2_PO_4_, 8 g of Na_2_HPO_4_ and 100 mL of 37% formaldehyde solution. Adjust the pH to 7.4 by using NaOH or HCl. If the pH remains stable after 20 min, add distilled water to a final volume of 1000 mL. Filter the solution and store it at 4 °C. The perfusion solution should be prepared as freshly as possible.

#### 2.9.7. Tris-EDTA Buffer Solution

Dissolve 6.05 g of Tris and 1.85 g of EDTA in 4500 mL of distilled water. Stir until fully dissolved, and adjust the pH to 9 by adding NaOH or HCl. If the pH remains stable after 20 min, add distilled water to a final volume of 5000 mL.

### 2.10. Wet Lab Procedures

#### 2.10.1. Transcardial Perfusion and Fixation

Anesthesia and monitoring:

Timing: 5 min.

(1) Anaesthetize each mouse with 0.1 mL per 10 g of body weight of a mixture consisting of 75% (*v*/*v*) ketamine and 25% (*v*/*v*) xylazine, administered via intraperitoneal injection.
(2) After one minute, assess the depth of anesthesia using the following procedures:
  (i) Pinch the base of the mouse’s tail with atraumatic forceps and observe any reactions. If no reactions are observed, proceed with the next step.
  (ii) Pinch the interdigital webbing of a foot with atraumatic forceps and observe any reactions. If no reactions are observed, proceed with the next step.
  (iii) Moisten the animals’ eyes with a small amount of ethanol and observe any reactions.

Caution: Proceed with the protocol only if the mouse does not react to the individual test. If any reaction is observed, wait another 15 s and repeat the test until no reaction occurs.

Transcardial perfusion:

Timing: 20 min per animal.

(3) Fix the mouse in a supine position on a corkboard or in a wax dish by securing all four limbs with pins in maximal abduction. Moisten the mouse with 70% (*v*/*v*) ethanol to prevent the spread of loose hair during dissection.
(4) Carefully open the abdominal and peritoneal cavities using sharp scissors. Remove the ventral part of the ribcage to expose the heart.
(5) Make an incision in the right atrium to allow blood and perfusion solution to flow out.
(6) Insert a butterfly cannula with a cut-off tip into the left ventricle.

Critical Step: Improper cannula placement may result in insufficient perfusion, as indicated by perfusion solution entering the lungs and exiting via the trachea/nose/mouth; under these circumstances, the cannula must be repositioned immediately.

(7) Perfuse the animal with 20 mL of ice-cold PBS using a 20 mL syringe. The injection should be performed with constant pressure over approximately 2 min.

Critical Step: Ensure no air is injected. Beyond, all blood should be flushed out before starting transcardial perfusion with the perfusion solution. Monitor liver tissue discoloration to confirm successful blood washout.

(8) Perfuse the animal with 50 mL of perfusion solution, following the same procedure as in step 7.
(9) Repeat step 8 or inject an additional 50 mL of perfusion solution using an automatic perfusion system. After this stage, the entire mouse, including its tail, should be stiff.

Dissection and postfixation:

Timing: 12 h.

(10) Decapitate the mouse and make an incision through the dorsal soft tissue to expose the skull. Insert the tip of the scissors into the foramen magnum, carefully cut along the calvaria, and gently spread the skull to reveal the brain.

Caution: At this stage, the brain tissue is highly fragile; therefore, handling should be kept to an absolute minimum until postfixation is complete to prevent or at least minimize tissue damage.

(11) Submerge the entire head in 30 mL perfusion solution and incubate at 4 °C for 12 h.

#### 2.10.2. Tissue Preparation and Paraffin Embedding

Timing: 13 h.

(12) Carefully extract the entire brain from the skull, place it in a histological sample container and rinse it in tap water for 6 to 12 h at room temperature. Trim off the olfactory bulb using a razor blade to expose the ventricular system, ensuring it will be properly filled with paraffin.
(13) Incubate the extracted brains in a series of solutions as specified in Table 1.

(14) Position the paraffin-soaked brains upright in small plastic vessels, resting on their rostral end, and carefully immerse them in molten paraffin at 60 °C. Secure the base of the histological container to the filled plastic vessel using additional paraffin.
(15) Allow the blocks to cool down for several hours or overnight, then carefully remove the plastic cap using a razor blade.
(16) Using a microtome, section the paraffin block at a thickness of 5 µm. Float the sections onto microscope slides in a room-temperature water bath, then transfer them to a 50 °C water bath to remove wrinkles and ensure optimal section flattening.
(17) Let the sections air dry at room temperature for at least 4 h, then incubate them at 40 °C overnight.

#### 2.10.3. Immunohistochemistry

Timing: 2 days.

Deparaffinization:

(18) Incubate the microscope slides in a series of solutions according to Table 2.

Antigen retrieval:

(19) Place the microscope slides in a heat-resistant vessel, ensuring they are fully submerged in the appropriate buffer (Table 3).

(20) Heat the buffer in a microwave set to 700 W until it reaches a boil.

Caution: Overheating may cause bubbles to dislodge the tissue from the slides.

(21) Reduce the power of the microwave to 200 W and allow the samples to simmer for another 10 min.
(22) Remove the vessel from the microwave and let it slowly cool to room temperature.
(23) Wash the sections by immersing them in PBS for 5 min under gentle agitation (ca. 120 rpm). Repeat this step 2 times.

Blocking:

(24) Carefully dry the microscope slides around the sections using tissue paper.
(25) Pipette 50 µL of 50% (*v*/*v*) NGS onto each section, ensuring complete coverage.
(26) Incubate the slides horizontally in a dark, humid chamber at room temperature for one hour.
(27) Wash the sections by immersing them in PBS for 5 min under gentle agitation (approx. 120 rpm). Repeat this step 2 times.

Caution: The sections must be kept continuously hydrated and must not be allowed to dry out at any point.

Primary antibody incubation:

(28) Carefully remove excess liquid using a paper towel.
(29) Dilute the primary antibody in NGS, as specified in Table 4.

Caution: Ensure through mixing of all solutions to achieve uniform staining.

(30) Pipette 50 µL of primary antibody solution onto each section, ensuring complete coverage.
(31) Incubate the sections overnight at 4 °C in a humid chamber.
(32) Wash the sections by immersing them in PBS for 5 min under agitation (approx. 120 rpm). Repeat this step 2 times.

Caution: Ensure that the stream from the pipette containing the antibody solution is not applied directly onto the section but slightly adjacent to it. This helps to avoid mechanical damage to the section during the staining procedure.

Peroxidase block:

(33) Incubate the microscope slides in PBS containing 0.3% (*v*/*v*) H_2_O_2_ for 30 min at room temperature, protected from light and under gentle agitation (approx. 120 rpm).
(34) Wash the sections by immersing them in PBS for 5 min. Repeat this step 2 times.

Secondary antibody incubation:

(35) Remove excess liquid using a paper towel.
(36) Cover each section with the appropriate Dako EnVision+ System—HRP (horse radish peroxidase) labelled Polymer Table 5.

(37) Incubate the sections in a humid chamber at room temperature for one hour.
(38) Wash the sections by immersing them in PBS for 5 min. Repeat this step 2 times.

Visualization:

(39) Incubate all slides in DAB (prepared according to the manufacturer) for 10 min.

Toxic: DAB is carcinogenic and teratogenic; handle with care.

(40) Rinse the sections briefly with tap water.
(41) Incubate the slides in distilled water for 5 min.

Nuclear counterstaining:

(Perform only with anti-GFAP-stained sections.)

(42) Submerge microscope slides in Mayer’s Hematoxylin working solution for 75 s.

Troubleshooting: The intensity of counterstaining may vary between batches. It is highly recommended to test the conditions on one or more sample slides before proceeding.

(43) Rinse the slides briefly in 1% (*v*/*v*) hydrochloric acid–ethanol mixture.
(44) Place the slides under a constant tap water flow for 5 min at room temperature.
(45) Incubate slides in distilled water for 3 min.

Dehydration and mounting:

(46) Incubate the stained sections in a series of solutions according to Table 6.

(47) Cover the sections with DePeX mounting medium and place cover slips on them, before letting them dry for at least 12 h at room temperature.

### 2.11. Dry Lab Procedures

#### 2.11.1. Microscopy

(48) Acquire histological images (RGB color model) of the ROI (in this project, the stratum oriens of the hippocampus) as tile scans in Z-stacks. Ensure that the entire thickness of the section is encompassed within the z-stack acquisition. In this project, a Leica DM6 B microscope equipped with the Leica DMC 6200 camera (Leica Microsystems CMS GmbH Wetzlar, Germany; 63-fold objective with oil, Leica Application Suite X software [version 3.7.0.20979, 2019, Germany]) was used. Generate a maximum projection.

#### 2.11.2. Cell Selection

(49) Label all GFAP^+^ cells with consecutive numbers, ensuring they meet the following criteria:
  (i) Located in the stratum oriens, between the corpus callosum and the pyramidal layer (Figure 4a).
  (ii) No overlap with blood vessels or other cells, to make sure that only individual cells are analyzed.
  (iii) A GFAP^+^ or hematoxylin-stained soma/nucleus must be clearly visible.
(50) Randomly select ten cells per animal from the total number of identified GFAP^+^ cells using, for example, the function “sample(x,10)” in R Studio, where x represents the total number of GFAP^+^ cells per animal.

#### 2.11.3. Digital Cell Reconstructions

(51) (A) Reconstruction with Neurolucida^®^ 360:
  (i) Open Neurolucida® 360, load a microscopic image via drag-and-drop and set/ensure the correct image scaling.
  (ii) Close the “3D Environment” window.
  (iii) Trace the soma (Figure 1f): Click >Trace>Neuron>Cell body< and mark the edges with left-clicks. Adjust line thickness by scrolling the mouse while tracing. Complete the tracing with a right-click and select >finish cell body<.
  (iv) Save the image with >File>Save as>Data File<.
  (v) Open the “3D Environment” window (>View>3D Environment<).
  (vi) Trace the cell processes using one of the following tracing modes (Figure 1f,g) (>Tree>Tracing Mode<):
    (a) “Smart manual”: Start tracing at the soma, follow the processes with left-clicks to their distal end. Adjust process thickness by pressing >Ctrl< and using the mouse wheel. Mark process endings with a right-click and select >Ending<.
    (b) “User-guided”: Start tracing at the soma and follow processes with left-clicks. Mark process endings with a right-click.
  (vii) Ensure branches belonging to the same process are connected. Modify individual points using >Tree>Edit>Points<.
  Caution: It is possible that the software automatically generates small, barely visible segments that in fact belong to a continuous cellular process. Because such erroneous segmentation can substantially bias the results, careful inspection and, if necessary, manual correction are essential.
  (viii) Set the process origin by selecting the proximal starting point and choosing “Set Point as Origin”. Click on >Tree>Edit>Points<, select the point that marks the proximal start of the process, and click on “Set Point as Origin”. If the origin is already set, “Selected point is already the origin of the tree” will be shown.
  (ix) Exit the edit menu via >Edit< and click on the symbol next to >Edit< on its left side.
  (x) Select the soma and all processes of a cell by pressing >Ctrl< and “right-click” and create a set with “Place Selected into Set”. Use logical designations like AC1 or cell1 as a set name.
  (xi) Save the file again.

(52) (B) Reconstruction with the SNT (Figure 1h):
  (i) iOpen ImageJ and the image you want to analyze.
  (ii) iiApply a Gaussian blur with >Process>Filter>Gaussian Blur<, setting “Sigma (Radius)” to 3.
  (iii) iiiDuplicate the image with > “Shift” + “D”.
  (iv) ivOpen SNT and press “OK” to convert RGB images to multichannel images.
  (v) vUncheck >Enable Snapping<.
  (vi) viChoose the “A*Search-Algorithm”. Trace the edges of the soma by “left-clicking”. Note that after every click, you must either confirm the newly created segment by pressing “Y” or deny it by pressing “N”. The completed path is ultimately confirmed with “F”. Tag the tracing as “Soma” via >SNT>Path Manager>Tag>Soma<.
  (vii) viClose the current image and invert the duplicated image from step 47 iii via clicking >Edit>Invert<.
  (viii) viiiOpen the image in the SNT via >File>Choose Tracing Image>From Open Image<.
  (ix) ixTrace dendrites beginning from a fork point at the soma. Therefore, select the soma tracing and move the cursor; possible fork points will be displayed. Then, create a path from that point to the point where the process branches. Confirm the segment by pressing “Y” and trace the emerging segments.
  (x) xTag all the process segments as dendrites: >SNT>Path Manager>Tag>Type>Dendrite (Basal)<.
  (xi) xiSave the tracings by >SNT>File>Save Tracings>Save as<.

#### 2.11.4. Quantification

(53) (A) Quantification with Neurolucida^®^ 360:
  (i) Open the Neurolucida^®^ Explorer.
  (ii) Go to >File>Preferences>Select Ordering< and select >Centrifugal ordering<.
  (iii) Use the >Batch analysis< function, select the relevant data files and click on >Open<.
  (iv) Check the boxes for the following analysis: “Segment”, “Segment points”, “Cell body”, and “Convex Hull”.
  (v) Also, check the boxes for >Separate by set< and >One Excel file for each data file< and finally click >Run<.
(54) (B) Quantification with the SNT:
  (i) Open the cellular model in the SNT and select all paths in the path manager.
  (ii) Click on >SNT>Path Manager> Analyze>Measurements>Measure Path(s)< and check the boxes for: “Length”, “No. of branch points”, and “Path order”. Start the analysis with >OK<.
  (iii) Save the results.

#### 2.11.5. Data Organization and Processing

(55) Combine the results to allow better handling of the data.

Caution: The analysis used in SNT defines the soma as a first-order process. If the given process orders in the SNT are compared to results from Neurolucida^®^ Explorer, they must be subtracted by one.

(56) Calculate the area of single-segment points with the parameters from the “Segment points” analysis using the following formula:

Area = ((“Start Diameter(µm)” + “End Diameter(µm)”)/2) × “Length(µm)”

Furthermore, calculate the total area per process and cell.

Caution: Since SNT cannot adequately measure the area of reconstructed processes, this step and the following 2 steps apply only to data obtained using Neurolucida^®^ 360.

(57) Calculate the total cell area by summing the soma area (from the “Cell body” analysis) and the area of the processes.
(58) Determine the ramification index by dividing the total cell area by the projection area (from the “Convex Hull” analysis).

### 2.12. Statistics

The resulting data sets were combined, organized and analyzed using R Studio [version 4.3.1; 2023-06-16 ucrt] [59,60,61,62,63,64,65,66,67,68,69,70,71]. To display the multidimensional dataset, a PCA with the “factoextra” package [72] was performed. Components with zero variance were excluded from the PCA. For the comparisons of single parameters, the data were tested for normal distribution and variance homogeneity with quantile–quantile plots and Levene tests. Depending on the samples, an ANOVA (analysis of variance) with a Tukey post-hoc test or a Kruskal–Wallis and Dunn’s test with Bonferroni *p*-value adjustment was performed. For the comparison of reconstruction-time and morphology parameters between Neurolucida^®^ 360 and the SNT, a paired *t*-test and a Wilcoxon signed-rank test were performed. Before that, the intraclass correlation coefficients for those groups were determined. Depending on the analytical method used, results were reported either as mean (M) with standard deviation (SD) or as median (Mdn) with mean absolute deviation (MAD).

## 3. Results

### 3.1. Cuprizone Intoxication Causes Demyelination and Microglial Activation

**Figure 2:**
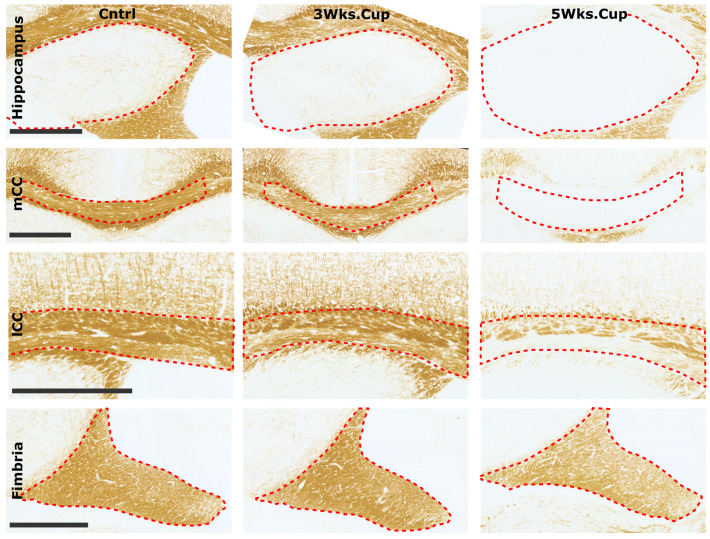
Representative images of anti-PLP-stained sections of control brains (Cntrl) and brains of mice that were intoxicated with cuprizone for either 3 weeks (3Wks.Cup) or 5 weeks (5Wks.Cup). The following brain regions are shown: first row, hippocampus; second row, medial corpus callosum (mCC); third row, lateral corpus callosum (lCC); fourth row, fimbria hippocampi. The red dotted lines demarcate the individual regions. Scalebars = 500 µm.

**Figure 3:**
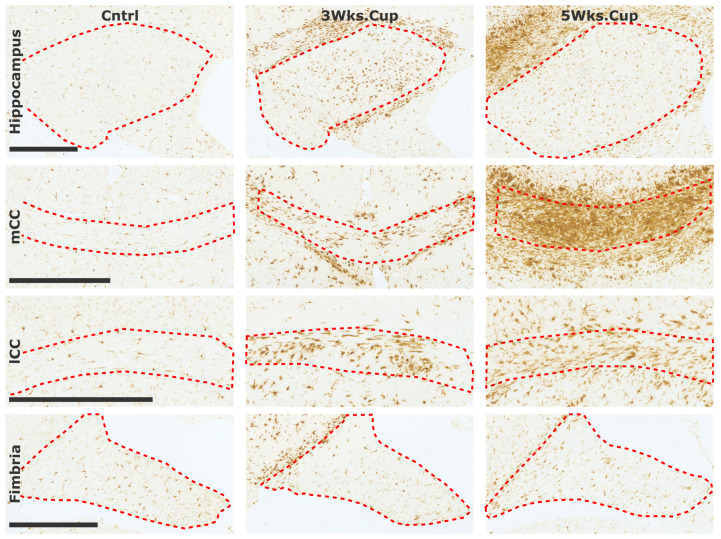
Representative images of anti-IBA1-stained sections of control brains (Cntrl) and brains of mice that were intoxicated with cuprizone for either 3 weeks (3Wks.Cup) or 5 weeks (5Wks.Cup). The following brain regions are shown: First row, hippocampus; second row, medial corpus callosum (mCC); third row, lateral corpus callosum (lCC); fourth row, fimbria hippocampi. The red dotted lines demarcate the individual regions. Scalebars = 500 µm.

To confirm cuprizone-induced demyelination, brain sections were immunohistochemically stained to visualize different antigens. Anti-PLP staining revealed robust myelination in control samples, with intense staining observed in the fimbria (Figure 2) and lighter staining within the cornu ammonis region of the hippocampus. After 3 weeks of cuprizone intoxication, a slight reduction in anti-PLP staining intensity was noted, particularly in the medial corpus callosum and the entire hippocampus formation, indicating the beginning of myelin loss. After 5 weeks, nearly complete demyelination was evident in the medial corpus callosum and hippocampus, accompanied by significant myelin loss in the lateral corpus callosum (lCC). The hippocampal fimbria showed just a minor loss of anti-PLP staining intensities. Analysis of anti-IBA1 staining showed sparse, individually distributed anti-IBA1-positive cells throughout the entire brain section in control animals (Figure 3). After 3 weeks of cuprizone intoxication, an increase in anti-IBA1 staining intensity was observed, especially within the midline of the corpus callosum (mCC) and the stratum oriens of the hippocampus formation, suggesting microglial activation. In the 5Wks.Cup group, the anti-IBA1 staining was further increased, particularly in the medial corpus callosum.

### 3.2. Astrocytes in the Cuprizone Model

**Figure 4:**
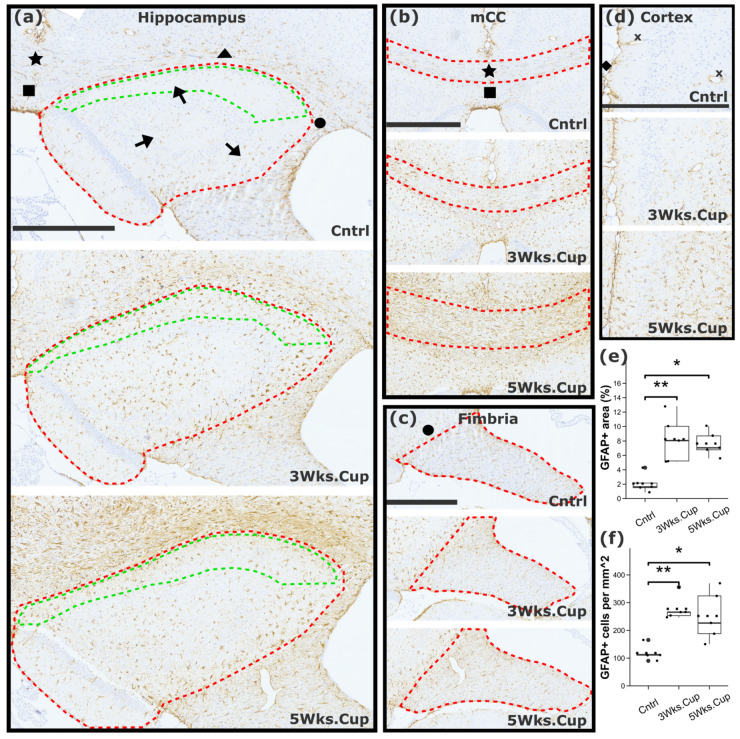
Representative images of anti-GFAP-stained sections of control brains (Cntrl) and brains of mice that were intoxicated with cuprizone for either 3 weeks (3Wks.Cup) or 5 weeks (5Wks.Cup). The following brain regions are shown: (**a**) hippocampus, (**b**) medial corpus callosum (mCC), (**c**) fimbria hippocampi and (**d**) neocortex. The red dotted lines in (**a**) demarcate the entire hippocampus; the green dotted lines show the region of interest (ROI) in the stratum oriens. The red dotted lines in (**b**) demarcate the midline of the corpus callosum. The red dotted lines in (**c**) demarcate the fimbria hippocampi. (★ mCC; ▲ lCC; ■ fornix ● alveus, glia limitans superficiales (◆) and perivascularis (x)), Scalebars = 500 µm. (**e**) The fraction of GFAP^+^ area and the number of GFAP^+^ astrocytes per mm^2^ in the striatum oriens. (**f**) Number of GFAP^+^ cells per mm^2^ in the ROI. ANOVA with Tukey’s test (dotted lines indicate the mean value), * *p* ≤ 0.05, ** *p* ≤ 0.01.

After confirming that cuprizone intoxication in this cohort of experimental animals induced the expected histopathological changes, namely demyelination and microglial activation, we next assessed astrocytic responses. To this end, brain sections were processed for anti-GFAP immunohistochemistry. As shown in Figure 4, in control animals, GFAP^+^ elements were detected in several major white matter tracts, including the corpus callosum, hippocampal fimbria, alveus, and the dorsal fornix, the latter located directly beneath the midline of the corpus callosum. In the neocortex of control animals, GFAP immunoreactivity was sparse and primarily restricted to the brain surface and perivascular regions, corresponding to the glia limitans superficialis and perivascularis, respectively (Figure 4d). GFAP^+^ cells were also visible in distinct hippocampal grey matter regions (see arrowheads in Figure 4a). Anti-GFAP immunostaining was visibly increased in both the 3 and 5Wks.Cup groups compared with controls.

To quantify GFAP expression specifically within the stratum oriens of the hippocampus (demarcated by the green dashed line in Figure 4a), the percentage of the total ROI area occupied by anti-GFAP-positive signal was determined. This analysis revealed a significant increase in GFAP expression after 3 weeks (M = 8.25%, SD = 3.27%, *p* ≤ **) and 5 weeks of cuprizone intoxication (M = 7.65%, SD = 0.08%, *p* ≤ *) compared with controls (M = 2.09%, SD = 0.13%) (Figure 4e).

Beyond, we quantified the number of GFAP^+^ cells per mm^2^ (id est, cellular density) in the hippocampal stratum oriens. As shown in Figure 4f, the density of GFAP^+^ cells per mm^2^ was significantly increased after 3 weeks (M = 277.74 cells/mm^2^, SD = 44.52 cells/mm^2^, *p* ≤ **) and 5 weeks of cuprizone intoxication (M = 252.01 cells/mm^2^, SD = 92.31 cells/mm^2^, *p* ≤ *) relative to the control group (M = 118.22 cells/mm^2^, SD = 27.99 cells/mm^2^).

### 3.3. Morphology of GFAP Expression

After having verified astrocyte activation in the hippocampal stratum oriens, digital models of individual anti-GFAP^+^ cells were constructed and diverse cellular geometric parameters were computed as described above.

**Figure 5:**
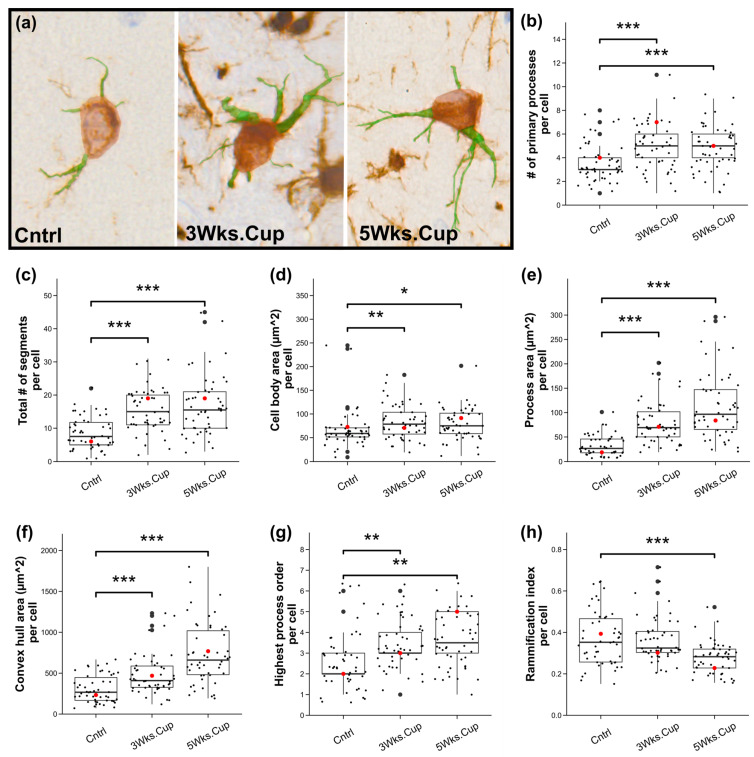
(**a**) In Neurolucida^®^ 360 reconstructed astrocytes. Comparison on the single-cell level between astrocytes from Cntrl, 3Wks.Cup and 5Wks.Cup animals according to their number of primary process segments (**b**), total number of process segments (**c**), cell body area (**d**), process area (**e**), convex hull area (**f**), highest process order (**g**), and the ramification index (**h**). Cells from (**a**) are visualized in the plots via red dots; Kruskal–Wallis test with Dunn’s post hoc test; * *p* ≤ 0.05, ** *p* ≤ 0.01, *** *p* ≤ 0.001, # = number.

As illustrated in Figure 5, the number of primary GFAP^+^ processes emerging from the astrocyte soma was significantly higher in both the 3Wks.Cup (*p* ≤ ***, Mdn (median) = 5, MAD (mean absolute deviation) = 1.5) and the 5Wks.Cup group (*p* ≤ ***, Mdn = 5, MAD = 1.5) compared with controls (*p* ≤ ***, Mdn = 3, MAD = 1.5) (Figure 5b). In line with this finding, the total number of process segments per cell was significantly increased in both cuprizone-intoxication groups (*p* ≤ ***) (Cntrl: Mdn = 7.5, MAD = 5.19; 3Wks.Cup: Mdn = 15, MAD = 5.93; 5Wks.Cup: Mdn = 15.5, MAD = 8.15), suggesting increased bifurcation (Figure 5c).

Numerous studies have demonstrated that astrocyte activation is morphologically accompanied by cellular hypertrophy [34,45,73]. We therefore next investigated whether the anti-GFAP-positive staining area, used here as a surrogate marker of astrocytic cell volume, is increased in the cuprizone model. To differentiate between hypertrophy of the astrocytic soma and hypertrophy of astrocytic processes, both parameters were analyzed separately.

As demonstrated in Figure 5d, cuprizone intoxication led to a significant enlargement of the cell body area in both the 3Wks.Cup group (*p* ≤ **, Mdn = 78.5 µm^2^, MAD = 35.7) and the 5Wks.Cup group (*p* ≤ *, Mdn = 75.0 µm^2^, MAD = 33.1) compared with controls (Mdn = 58.8 µm^2^, MAD = 15.88). Comparable to what we found for the cell soma, the total process area was significantly increased in the 3Wks.Cup (*p* ≤ ***, Mdn = 69,13 µm^2^, MAD = 36,69 µm^2^) and 5Wks.Cup (*p* ≤ ***, Mdn = 96.56 µm^2^, MAD = 62.64 µm^2^) groups, indicating an overall upregulation of GFAP expression compared to the Cntrl group (Mdn = 26.88 µm^2^, MAD = 22.62 µm^2^) (Figure 5e). Together, the increases in soma size and process area point to cellular hypertrophy in response to the cuprizone-induced demyelination.

It has been shown previously that astrocytes, even under hypertrophic conditions, do not significantly change the territory of the entire cell [34,35,45,73]. To address this aspect, the astrocytic projection areas covered by GFAP^+^ processes (i.e., convex hull) were analyzed. As demonstrated in Figure 5f, the convex hull area was significantly enlarged after 3 weeks (*p* ≤ ***, Mdn = 409.089 µm^2^, MAD = 168.36 µm^2^) and 5 weeks (*p* ≤ ***, Mdn = 660.13 µm^2^, MAD = 315.40 µm^2^) of cuprizone intoxication compared to controls (Mdn = 266.88 µm^2^, MAD = 169.44 µm^2^), suggesting an expansion of the GFAP^+^ cellular territory. In line with this finding, the value of the highest process order was significantly higher in the 3Wks.Cup and 5Wks.Cup groups compared to controls. More in detail, the highest process order was Mdn = 2 (MAD = 1.5) in control astrocytes, Mdn = 3 (MAD = 1.5, *p* ≤ **) in the 3Wks.Cup and Mdn = 3.5 (MAD = 2.2, *p* ≤ **) in 5Wks.Cup group (Figure 5g).

Another approach to quantify cellular complexity is the ramification index, defined as the ratio of the total cell area to the convex hull area. In highly ramified cells with extensive processes, the ramification index is low (<1), whereas rounded cells lacking projections exhibit values approaching one. As demonstrated in Figure 5h, compared to controls, the ramification index was, by trend, lower at week 3 (Mdn = 0.32, MAD = 0.07), and significantly lower (*p* ≤ ***) at week 5 of cuprizone intoxication (Mdn = 0.28, MAD = 0.07) (Cntrl: Mdn = 0.35, MAD = 0.14).

### 3.4. Heterogeneity During Reactive Astrogliosis

**Figure 6:**
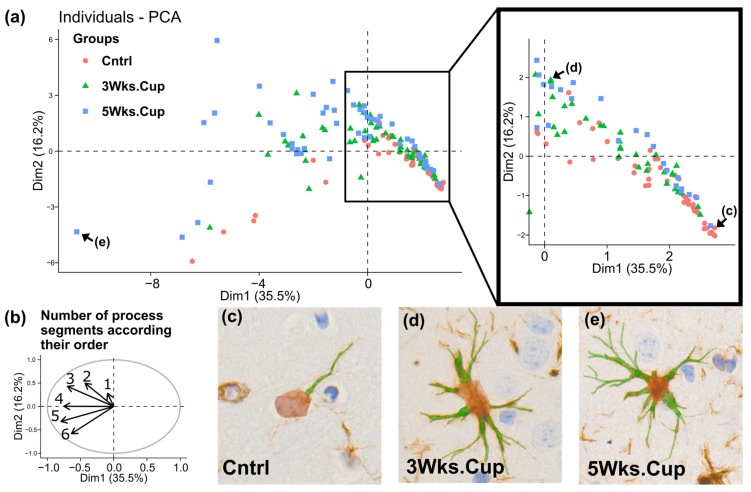
Principal component analysis (PCA) of all reconstructed astrocytes, considering the number, length and area of process segments according to their order. Principal component analysis (PCA) for individuals (**a**) and loadings plot with the variable “number of processes” (**b**) is shown. In (**b**), numbers 1 to 6 indicate the different segment orders. The loadings plots “medium process length” and “medium process area” are shown in the supplements. (**c**,**d**,**e**) Representative anti-GFAP^+^ cells from control and 3 weeks and 5 weeks cuprizone-intoxicated mice. The locations of these individual cells on the PCA plot (**a**) are highlighted by arrows.

To visualize phenotypical heterogeneity and group-specific differences among reconstructed cells, PCA was performed using the values for mean process length, mean process area, and the number of process segments across centrifugal process orders 1–6 (Figure 6a). To not compromise the informativeness of the PCA, convex hull and ramification index were not included because their dependence on the cell area could have resulted in redundancies. In the PCA plot of individuals (see Figure 6a), spatial proximity of data points reflects morphological similarity. On the other hand, the spatial distance of data points reflects morphological differences. In line with this, data points of the cells shown in Figure 6c–e, which exhibit clear phenotypical differences, are positioned far apart from each other in the plot.

A majority of control cells were located closely together in the lower right quadrant, indicating shared structural features (Figure 6a). In contrast, cells from 3Wks.Cup and 5Wks.Cup groups were broadly dispersed throughout the PCA space, reflecting increased phenotypical heterogeneity. Notably, a subset of anti-GFAP^+^ cells of cuprizone-intoxicated mice co-localized with cells from the Cntrl group, suggesting that these astrocytes still share similarities in their GFAP topology despite ongoing demyelination.

The loadings plots from this PCA are shown in Figure 6b and Figure S1a,b. It can be noted that the variables (number of processes, process length and process area) of the same process order are oriented together and show similar representation in the PCA (Figure S1c). Increasing the distance of an individual data point from the origin in the direction of a variable indicates a stronger influence of this variable on the data point/cell.

Therefore, the majority of cells from the Cntrl group were primarily characterized by a relative scarcity of higher-order branches. In contrast, cells from 3Wks.Cup and 5Wks.Cup showed stronger associations with higher-order branching variables, consistent with increased bifurcation.

Together, these PCA results suggest that the observed group differences, as demonstrated in Figure 5, are due to inter- as well as intragroup differences.

### 3.5. Comparison of Neurolucida^®^ 360 and the SNT

**Figure 7:**
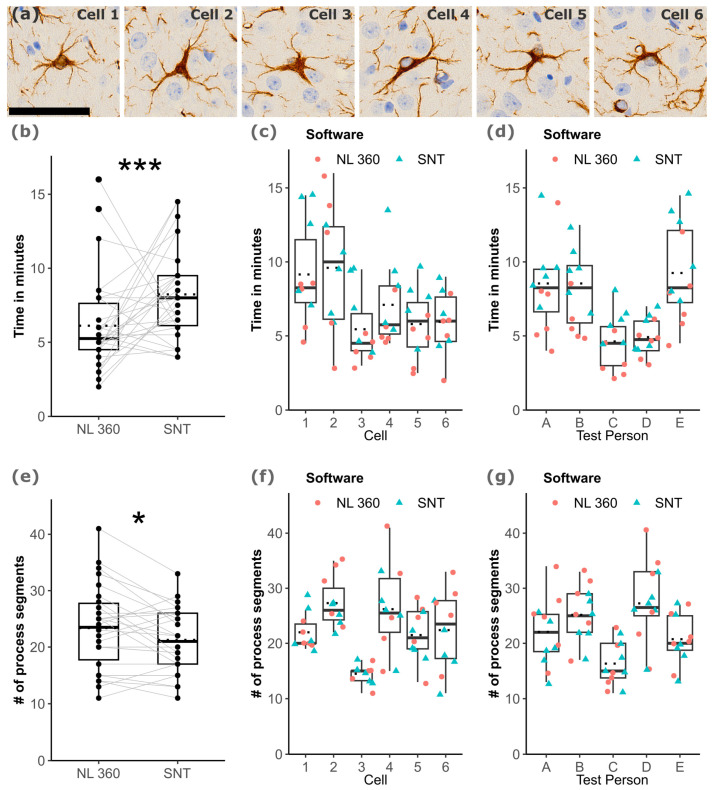
(**a**) Individual anti-GFAP-labeled astrocytes that were reconstructed by the different test persons. Scalebar = 50 µm. Reconstruction time (**b**–**d**) and number of reconstructed process segments (**e**–**g**) in relation to program, test subject, and sample cell being reconstructed ((**b**) Wilcoxon signed-rank test; (**e**) paired *t*-test). Dotted horizontal lines represent the mean value. Gray lines indicate pairs of sample cells, reconstructed by the same test person. * *p* ≤ 0.05, *** *p* ≤ 0.001, # = number.

So far, we have used the commercial cellular reconstruction tool Neurolucida^®^ 360. The SNT plugin offers an open-source alternative. To compare their practicability, six test persons reconstructed six astrocytes each using both tools (Figure 7). It was found that the reconstruction of a single cell took a person, on average, 6.1 min using Neurolucida^®^ 360 and 8.2 min using the SNT (Figure 7b). Statistical comparison using the Wilcoxon signed-rank test confirmed that this difference was significant (*p* ≤ ***). Additionally, significant variations in reconstruction times were observed between cells (Figure 7c). While cell no. 3, with only a few processes, was reconstructed within an average of 5.5 min (combining times from Neurolucida^®^ 360 and the SNT), cell no. 2, which exhibited greater bifurcation with many small processes, was reconstructed in an average of 9.6 min (Figure 7f). Furthermore, variations in reconstruction times were observed between test persons (Figure 7d). The fastest test person (C) completed a cell reconstruction in an average of 4.6 min, while the slowest (E) required 9.3 min. As a surrogate marker for reconstruction precision, the total number of segments per cell was assessed. In total, participants using Neurolucida^®^ 360 detected 701 segments, whereas those using the SNT detected 638 segments. From a total of 30 reconstructions (six cells times five operators), in only seven instances did a test person find more segments with the SNT than with Neurolucida^®^ 360 (Figure 7e). A paired *t*-test showed that the test persons reconstructed significantly more segments with Neurolucida^®^ 360. Furthermore, substantial differences were observed among test persons (Figure 7g). While test person C detected 196 total segments, test person D detected 327.

To further evaluate potential software-dependent differences, additional branching parameters were compared (Figure S2). The number of primary processes per cell was slightly but significantly higher when reconstructions were performed using the SNT compared with Neurolucida^®^ 360 (Figure S2a, *p* ≤ *). Likewise, the mean segment length per cell was significantly increased in SNT-based reconstructions, indicating a tendency toward longer traced process segments (Figure S2d, *p* ≤ *). In contrast, the highest process order, reflecting branching hierarchy and arbor complexity, was markedly lower in SNT reconstructions than in Neurolucida^®^ 360 (Figure S2g, *p* ≤ ***).

In summary, Neurolucida^®^ 360 enabled faster reconstructions and yielded a higher number of detected segments, especially finer ones, compared with the presented workflow with the SNT plugin. Nevertheless, reconstruction time and output varied substantially depending on cell complexity and test person.

## 4. Discussion

Cells exhibit a remarkable diversity of shapes that are closely linked to their functions. Flat, cuboidal or columnar cells optimize surface contacts in epithelia, whereas elongated or spindle-shaped cells (e.g., fibroblasts, muscle cells) facilitate migration and contraction. In the CNS, neurons and glia display complex morphologies: neurons are highly branched for signal integration and transmission, while glial cells have specialized shapes for support and immune roles. For example, cortical pyramidal neurons have two polarized dendritic arbors (apical and basal) that receive distinct inputs [74], and cerebellar Purkinje cells possess planar, fan-shaped arbors aligned to intersect parallel fibers. Astrocytes form a “bushy”, star-like network of fine processes contacting synapses and blood vessels. Microglia in a healthy brain are highly ramified with small somata for surveillance, whereas in injury or disease, they retract processes and become hypertrophic or ameboid for phagocytosis. Oligodendrocytes have small somata and extend a few processes, each producing multiple myelin internodes on nearby axons. These unique shapes enable CNS cells to fulfill functions such as synaptic integration, metabolic support, immune surveillance, and rapid signal conduction. In CNS disease, cell morphologies change markedly. For example, autism and related syndromes often feature abnormal neuronal branching and spine structure [75], and in Down syndrome mouse models, cortical projection neurons have fewer mature (mushroom-shaped) spines and more stubby spines compared to controls [76]. Regarding glia cells, under inflammatory conditions, microglia retract branches and enlarge somata (ameboid appearance), and astrocytes undergo hypertrophy, lose many fine processes and assume a scar-like morphology [77].

In this study, a quantitative experimental pipeline was established to assess phenotypical aspects of astrocytes based on the expression of one of the most important and abundant astrocytic intermediate filaments, namely GFAP. In contrast to traditional qualitative descriptors, this approach yields quantitative metrics that enable robust statistical comparisons between experimental groups and will enable correlations with other imaging modalities such as functional MRI. To study differences in topological intermediate expression under physiological and pathological conditions, sections from control and cuprizone-intoxicated animals were included in this study.

In a first step, we verified the successful induction of cuprizone-induced demyelination by investigating the myelin content as well as microglia activation. In line with several previous studies, profound demyelination was observable in the midline of the corpus callosum and the hippocampal grey matter, parallelled by the activation of microglia cells [49,78,79,80,81,82]. Further, we found a pronounced induction of GFAP expression levels, indicative of ongoing reactive astrocytosis [83,84].

After having confirmed the successful induction of demyelination, we evaluated GFAP topology using an unbiased approach. Quantitative analysis of anti-GFAP-labeled astrocytes revealed significant differences between control and cuprizone-intoxicated cells. Principal component analysis demonstrated tight clustering of control astrocytes and marked heterogeneity among reactive cells, reflecting extensive GFAP-network remodeling in accordance with the reported structural and functional diversity of reactive astrocytes [23].

In cuprizone-intoxicated mice, significant increases in total numbers of process segments and primary processes were observed, indicating enhanced branching. These findings are consistent with previous studies demonstrating increased process complexity [23]. Furthermore, we observed an enlargement of total process area and soma size, supporting the presence of astrocyte hypertrophy, as described previously in this model [85]. In addition, higher centrifugal process orders and enlarged GFAP^+^ convex hull areas were observed, indicating a distal expansion of GFAP immunoreactivity. Notably, this appears to contrast with previous reports demonstrating no significant alteration in total astrocytic projection area during reactive astrogliosis [35,85]. At this point, it is important to notice that in the present study, we focused specifically on the expression and spatial distribution of the intermediate filament protein GFAP in astrocytes. It is important to emphasize that GFAP represents only a subset of the astrocytic cytoskeleton and predominantly labels the major processes and filamentous scaffold of the cell. Fine distal processes, including perisynaptic astrocytic leaflets, are only partially captured by GFAP immunoreactivity and are instead more reliably visualized using alternative markers such as ALDH1L1 (Aldehyde Dehydrogenase 1 Family Member L1) [86]. The observed increase in total process area per astrocyte may, at least in part, reflect elevated GFAP protein expression at the single-cell level, thereby enhancing the detectability of pre-existing fine processes by anti-GFAP immunolabeling. This interpretation is further supported by our regional analyses, which demonstrate not only an expansion of the GFAP-positive area but also an increase in the density of GFAP-positive astrocytes.

Consequently, GFAP-based analyses do not comprehensively reflect the entire astrocytic territory or the full complexity of astrocyte morphology. Rather, our findings should be interpreted as evidence for a redistribution or remodeling of the intermediate filament network within astrocytes. While such changes may be associated with broader structural adaptations, direct conclusions regarding alterations of the total astrocytic morphology remain limited and would require complementary labeling strategies targeting the complete cellular architecture.

Several other methods exist to visualize the complexity of astrocyte morphology. Iontophoretic dye-filling, for example, allows detailed three-dimensional visualization of entire astrocytes [45]. Using this method, a fine glass micropipette containing a fluorescent dye (e.g., Lucifer Yellow or biocytin) is advanced toward an individual astrocyte under microscopic guidance. By applying a small, controlled electrical current, the negatively charged dye is driven into the targeted cell through the pipette tip. The dye subsequently diffuses throughout the cytoplasm, filling the soma and all cellular processes, including fine distal branches. After fixation and appropriate processing, the filled astrocyte can be imaged in three dimensions using confocal or two-photon microscopy, enabling high-resolution reconstruction of the entire cellular morphology. However, this technique is technically demanding and does not allow high-throughput screening [87]. Another option to visualize entire astrocyte cell bodies is the use of transgenic animals expressing fluorescent marker proteins such as GFP (green fluorescent protein) under the control of an astrocyte-specific promoter such as GFAP, ALDH1L1 or GLAST1 (GLutamate ASpartate Transporter 1) [86,88,89]. A major advantage of this approach is its cell-type specificity, as promoter-driven expression restricts labeling predominantly to astrocytes, thereby reducing ambiguity compared to non-selective dyes. Cytoplasmic fluorescent proteins can fill the soma and major processes, enabling three-dimensional reconstruction of astrocytes and, depending on the promoter and expression level, visualization of fine distal branches. In addition, this method is well-suited for longitudinal in vivo imaging, such as two-photon microscopy, allowing dynamic analyses of astrocytic structural plasticity over time. Because labeling is genetically encoded, it avoids invasive single-cell manipulation and enables the simultaneous analysis of large astrocyte populations with high reproducibility. Furthermore, reporter lines can be combined with immunohistochemistry, functional imaging, or optogenetic approaches. However, several limitations must be considered. The extent and pattern of labeling strongly depend on the chosen promoter, which may target distinct astrocyte subpopulations and exhibit regional or developmental variability. In addition, high levels of fluorescent protein expression could potentially influence cellular physiology, and the maintenance and crossbreeding of transgenic colonies increase logistical complexity and cost. Consequently, while transgenic reporter lines provide a robust and versatile tool for astrocyte visualization, careful interpretation is required with respect to promoter specificity and potential labeling biases [89]. Another elegant method to quantify phenotypical aspects in astrocytes has recently been described by Marques and colleagues [90]. In this study, the authors established a semi-automated workflow combining threshold-based segmentation, skeletonization, and branching analysis to extract quantitative parameters from bulk fluorescence images. The method enables objective assessment of astrocyte number, total process length, branching points, and branch order without the need for time-intensive manual tracing of individual cells. Importantly, it was designed to work on standard epifluorescence or confocal images of immunolabeled sections rather than requiring single-cell dye-filling or transgenic reporter lines. A key advantage is scalability: because the method operates on conventional bulk immunohistochemistry images, it allows analysis of large tissue areas and substantial cell populations, thereby increasing statistical power and improving representativeness compared to single-cell-filling approaches. However, segmentation accuracy might be influenced by staining quality, background signal, and image resolution; variations in these factors can affect thresholding and skeletonization outcomes. Additionally, bulk image analysis may struggle to reliably separate overlapping astrocytic processes in densely labeled regions, which can lead to merged skeletons and overestimation of branching complexity.

## 5. Conclusions

The protocol described in this study, although time-intensive, provides a high level of resolution of the GFAP network at the single-cell level. It enables detailed quantification of structural parameters such as primary branch number, higher-order branching patterns, and soma volume, thereby allowing a refined characterization of astrocyte architecture. Importantly, this approach offers the flexibility of integration with advanced imaging modalities in the future, including high-resolution confocal z-stacks or super-resolution techniques, which may further enhance three-dimensional reconstruction fidelity [91,92,93].

Our evaluation of available reconstruction software revealed additional methodological constraints that merit consideration. Open-source platforms, while highly valuable and accessible, lacked, in this case, certain analytical features (e.g., process area) and automation features that are available in commercial systems. Conversely, even in commercially available software, semi-automated reconstruction workflows remain labor-intensive and require substantial manual correction, particularly in densely branched or overlapping structures. As a consequence, reconstruction quality is partly dependent on user expertise, and the potential for operator-related variability cannot be fully excluded.

Despite these limitations, both workflows with open-source and commercial tools proved suitable for systematic astrocyte reconstruction and quantitative morphometric analysis. When applied with standardized acquisition parameters and clearly defined reconstruction criteria, they provide a robust framework for detailed structural assessment of astrocytes at the single-cell level.

## Figures and Tables

**Table 1:** Paraffinization of the tissue samples.

Step	Solution	Incubation Time	Comment
I.	Ethanol 50% (*v*/*v*)	overnight	incubate at 4 °C
II.	Ethanol 70% (*v*/*v*)	1 h	incubate under gentle agitation at room temperature
III.	Ethanol 70% (*v*/*v*)	1 h	incubate at 60 °C
IV.	Ethanol 80% (*v*/*v*)	1 h	incubate at 60 °C
V.	Ethanol 96% (*v*/*v*)	1 h	incubate at 60 °C
VI.	Ethanol 96% (*v*/*v*)	1 h	incubate at 60 °C
VII.	Ethanol 100% (*v*/*v*)	1 h	incubate at 60 °C
VIII.	Ethanol 100% (*v*/*v*)	1 h	incubate at 60 °C
IX.	Ethanol 100% (*v*/*v*)	1 h	incubate at 60 °C
X.	Xylene	1 h	incubate at 60 °C
XI.	Xylene	1 h	incubate at 60 °C
XII.	Xylene	1 h	incubate at 60 °C
XIII.	Paraffin	1 h	incubate at 60 °C
XIV.	Paraffin	1 h	incubate at 60 °C
XV.	Paraffin	1 h	incubate at 60 °C

**Table 2:** Solutions for deparaffinization.

Step	Solution	Incubation Time
I.	Xylene	10 min
II.	Xylene	10 min
III.	Xylene	10 min
IV.	Xylene/Ethanol (50%/50% (*v*/*v*))	5 min
V.	Ethanol 100% (*v*/*v*)	3 min
VI.	Ethanol 100% (*v*/*v*)	3 min
VII.	Ethanol 96% (*v*/*v*)	3 min
VIII.	Ethanol 96% (*v*/*v*)	3 min
IX.	Ethanol 70% (*v*/*v*)	3 min
X.	Ethanol 50% (*v*/*v*	3 min
XI.	Distilled Water	3 min

**Table 3:** Buffer selection for antigen retrieval.

Antigen	Supplier	Buffer
GFAP (Glial Fibrillary Acid Protein)	Abcam, Cambridge, UK (RRID: AB_1209224)	Tris-EDTA
IBA1 (Ionized Calcium-Binding Adaptor Molecule 1)	FUJIFILM Wako Pure Chemical Corporation, Osaka, Japan (RRID: AB_8395049)	Tris-EDTA
PLP (Proteolipid Protein 1)	Bio-Rad AbD Serotec GmbH, Neurid, Germany, ((RRID: AB_2237198)	Not applicable

**Table 4:** Antibody concentration.

Antigen	Supplier	Concentration
GFAP (Glial Fibrillary Acid Protein)	Abcam, Cambridge, UK (RRID: AB_1209224)	1:750
IBA1 (Ionized Calcium-Binding Adaptor Molecule 1)	FUJIFILM Wako Pure Chemical Corporation, Osaka, Japan (RRID: AB_8395049)	1:1000
PLP (Proteolipid Protein 1)	Bio-Rad AbD Serotec GmbH, Neurid, Germany, ((RRID: AB_2237198)	1:5000

**Table 5:** Secondary antibodies.

Antigen	Dako Envision+ System—HRP Labelled Polymer
GFAP (Glial Fibrillary Acid Protein)	Anti-Rabbit (K4003)
IBA1 (Ionized Calcium-Binding Adaptor Molecule 1)	Anti-Rabbit (K4003)
PLP (Proteolipid Protein 1)	Anti-Mouse (K4001)

**Table 6:** Solutions for dehydration and mounting.

Step	Solution	Incubation Time
I.	Ethanol 50% (*v*/*v*)	3 min
II.	Ethanol 70% (*v*/*v*)	3 min
III.	Ethanol 96% (*v*/*v*)	3 min
IV.	Ethanol 96% (*v*/*v*)	3 min
V.	Ethanol 100% (*v*/*v*)	3 min
VI.	Ethanol 100% (*v*/*v*)	3 min
VII.	Xylene/Ethanol (50%/50% (*v*/*v*))	5 min
VIII.	Xylene	10 min
IX.	Xylene	10 min
X.	Xylene	10 min

## Data Availability

The data presented in this study are available on request from the corresponding author.

## Supplementary Materials

The following supporting information can be downloaded at: https://www.mdpi.com/article/10.3390/cells15110964/s1, Figure S1: Principal component analysis (PCA). Loadings plots representing the mean length (a) and mean area (b) of processes segments according their order. c) Cos2-Quality of representation with the variables for the mean process length (“LengthMean”), mean process area (“meanArea”) and the number of process segments (“Process.numb”) according to their process segment order (digit behind the variable name).; Figure S2 Comparison between Neurolucida^®^ 360 and SNT according the number of reconstructed primary process segments, mean length of process segments and the highest reconstructed process order. Grey lines indicate pairs of sample cells, reconstructed by the same test person. Dotted horizontal lines indicate the mean value. Wilcoxon signed-rank test; * *p* ≤ 0.05, *** *p* ≤ 0.001, # = number.

## Abbreviations

The following abbreviations are used in this manuscript:

ALDH1L1	Aldehyde Dehydrogenase 1 Family Member L1
ANOVA	Analysis of Variance
ATP	Adenosine triphosphate
BBB	Blood–brain barrier
Ca^2+^	Calcium ion
CNS	Central nervous system
Cuprizone	Bis(cyclohexanone)oxaldihydrazone
DAB	3,3′-Diaminobenzidine
GFAP	Glial Fibrillary Acid Protein
GLAST1	GLutamate ASpartate Transporter 1
IBA1	Ionized calcium-binding adaptor molecule 1
IHC	Immunohistochemistry
lCC	Lateral corpus callosum
M	Mean
MAD	Median absolute deviation
mCC	Medial corpus callosum
Mdn	Median
MEGF10	Multiple Epidermal Growth Factor-Like Domains Protein 10
MERTK	MER Proto-Oncogene, Tyrosine Kinase
MRI	Magnetic Resonance Imaging
MS	Multiple sclerosis
NGS	Normal goat serum
PBS	Phosphate-buffered saline
PCA	Principal Component Analysis
PLP	Proteolipid protein 1
RI	Ramification index
SD	Standard deviation
SNT	Simple Neurite Tracer
