# Supplementary material for: Quantitative Assessment of GFAP-Based Astrocyte Morphology in the Cuprizone Model: A Comparative Evaluation of Neurolucida® 360 and SNT
Source: Cells. 2026 May 22;15(11):964. doi: 10.3390/cells15110964 (PMC13257050; doi:10.3390/cells15110964)
Supplement: Supplementary file 1 [file cells-15-00964-s001.zip › cells-4267136-supplementary.pdf]

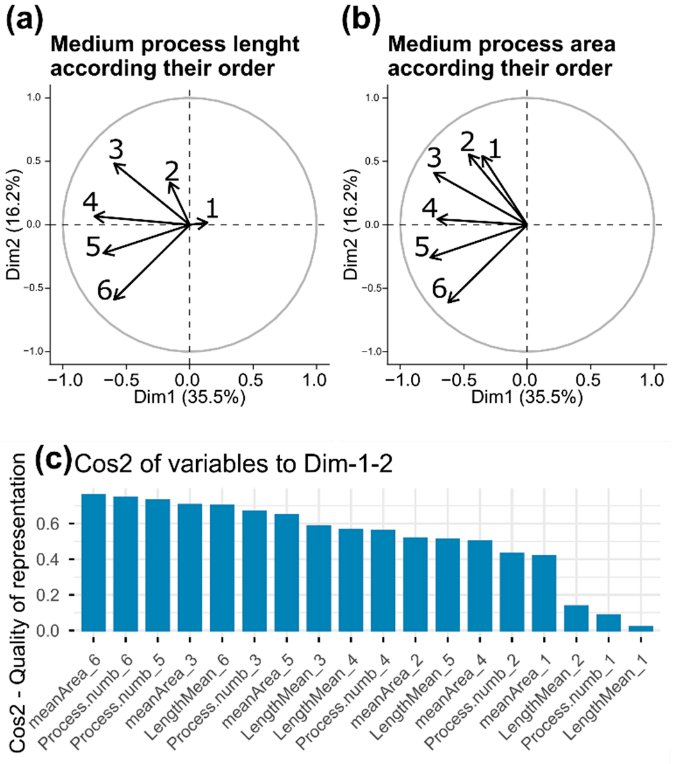

Figure S1 Principal component analysis (PCA). Loadings plots representing the mean length (a) and mean area (b) of processes segments according their order. c) Cos2-Quality of representation with the variables for the mean process length ("LengthMean"), mean process area ("meanArea") and the number of process segments ("Process.numb") according to their process segment order (digit behind the variable name).

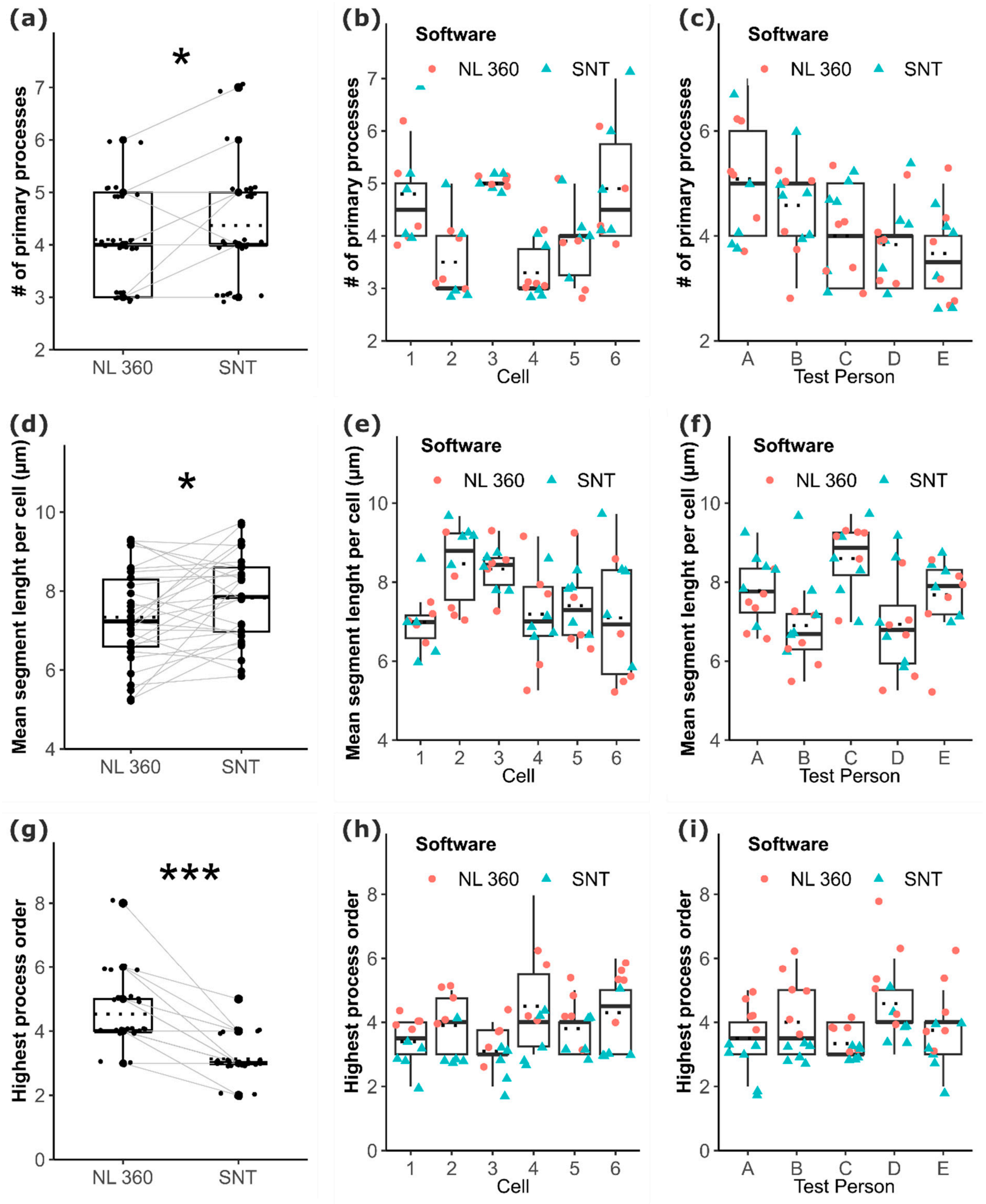

Figure S2 Comparison between Neurolucida 360® and SNT according the number of reconstructed primary process segments, mean length of process segments and the highest reconstructed process order. Grey lines indicate pairs of sample cells, reconstructed by the same test person. Dotted horizontal lines indicate the mean value. Wilcoxon signed-rank test; *p≤0.05, ***p≤0.001, # = number.
